# Prevalence and Interrelation of Irritable Bowel Syndrome With Generalized Anxiety Disorder Among Sudanese Medical Students, 2020

**DOI:** 10.1192/bjo.2024.169

**Published:** 2024-08-01

**Authors:** Samah H. Heamid, Danya Ibrahim

**Affiliations:** Khartoum University, Faculty of Medicine, Khartoum, Sudan

## Abstract

**Aims:**

This study aimed to determine the prevalence of irritable bowel syndrome (IBS) among medical students at Khartoum University and to examine its association with generalized anxiety disorder (GAD).

**Methods:**

This cross-sectional study was conducted between December 2020 and February 2021, using the Rome IV criteria to diagnose IBS and a 7-item generalized anxiety disorder (GAD-7) scale to assess GAD. A total of 395 self-administered questionnaires were distributed using proportional allocation based on percentages of students in each academic year and their gender. Simple random sampling was used to select participants. The analysis was done using SPSS, and a p-value of <0.05 was considered significant.

**Results:**

We included 325 medical students with a mean age of 21.4 ± 2.2 years, and 69.5% females and 30.5% males. The overall prevalence of IBS was 16.6%, with the most common subtype being IBS-M (35%), followed by IBS-D (31%), IBS-C (28%), and IBS-U (6%). The prevalence of GAD was 22.8%, and anxiety was detected in 54.5% of students. 7.7% of students had both IBS and GAD, and there was a statistically significant relationship between IBS and GAD (Chi-square = 20.385; p < 0.001).

**Conclusion:**

The study findings aligned with previous literature underscoring the prevalence of IBS and GAD among medical students at Khartoum University. Also, sheds light on a substantial association between them. Providing psychological support and stress management programs to medical students is paramount and key to a favorable prognosis.

